# Effect of Antiretroviral Therapy, Immune Dysregulation, and COVID‐19 Vaccination on SARS‐CoV‐2 Outcomes in PLWHA in Kenya

**DOI:** 10.1155/arat/5621772

**Published:** 2026-10-05

**Authors:** James Kinoti Njue, Lihana Wekesa, Alex Maiyo, Zakayo Maingi Mwangi, Dorothy Kagendo, Raphael Mwamtsi Lwembe

**Affiliations:** ^1^ School of Medicine Moi University Eldoret Kenya mu.ac.ke; ^2^ Centre for Virus Research (CVR) Kenya Medical Research Institute (KEMRI) Nairobi Kenya kemri.org; ^3^ Department of Medical Laboratory Science Meru University of Technology Meru Kenya; ^4^ School of Nursing and Public Health Chuka University Chuka Kenya chuka.ac.ke

**Keywords:** ART, COVID-19, HIV, immune dysregulation, Kenya, SARS-CoV-2, vaccination

## Abstract

**Background:**

People living with HIV (PLWHA) may experience increased vulnerability to SARS‐CoV‐2 infection and severe COVID‐19 due to immune dysregulation, comorbidities, and treatment adherence challenges. Kenya has over 1.4 million PLWHA. However, only a few have been reported with severe COVID‐19, suggesting potential protective roles of antiretroviral therapy (ART), management of comorbidities, and COVID‐19 vaccination.

**Methods:**

This study was conducted across high HIV‐prevalence regions in Kenya (Nakuru, Malindi/Mariakani, and Kisumu). A cross‐sectional survey evaluates clinical characteristics and self‐reported COVID‐19 vaccination outcomes among 303 PLWHA. Quantitative data were collected through structured questionnaires and clinical records. Associations between ART, immune markers (CD4/CD8 counts; viral load), COVID‐19 vaccination, and SARS‐CoV‐2 infection and severity were tested using Chi‐square and Fisher’s exact tests.

**Results:**

An analysis of 303 participants (70.6% female, median age 40) revealed an 86.5% COVID‐19 vaccination rate and 94.1% ART adherence. Only 9.2% reported prior SARS‐CoV‐2 infection, mostly mild, potentially indicating underreporting or asymptomatic cases. Interestingly, missing ART doses were significantly associated with contracting SARS‐CoV‐2 (*p* = 0.03). Furthermore, all participants reporting or demonstrating SARS‐CoV‐2 infection were vaccinated, compared to 85.1% of uninfected participants (*p* = 0.014). Due to incomplete health records, data for CD4 counts were available for only 7.6% of participants (*n* = 23), limiting conclusions regarding immune dysregulation.

**Conclusion:**

Low COVID‐19 rates were observed. All cases occurred in vaccinated participants, likely reflecting health‐seeking bias rather than vaccine failure. Missing ART doses were significantly associated with infection. Optimal risk mitigation requires integrated strategies prioritizing both high vaccine coverage and strict ART adherence.

## 1. Background

The onset of the Coronavirus Disease 2019 (COVID‐19) pandemic highlighted the need for understanding its impact on vulnerable populations such as people living with HIV/AIDS (PLWHA) [1]. Approximately 40 million people are living with HIV globally, with sub‐Saharan Africa bearing the highest burden of 70% of the cases [2–5]. Kenya has approximately 1.5 million PLWHA with antiretroviral therapy (ART) coverage reaching 80% of the diagnosed persons [2]. COVID‐19 is responsible for over 770 million infections and over 7 million deaths worldwide with sub‐Saharan Africa reporting relatively lower cases, although under‐reporting has been a concern. In Kenya, cumulative COVID‐19 cases were above 400,000 with more than 5000 deaths by late 2023 [2, 6, 7].

Literature shows heightened risk of severe COVID‐19 among PLWHA, notably among individuals with uncontrolled viral replication; diminished CD4 counts; or comorbid diseases such as diabetes, hypertension, tuberculosis, and cancer [8–11]. However, data remain unclear with other studies reporting that ART, immune regulation, and vaccination may confer protection against SARS‐CoV‐2 infection or reduce disease severity [12–15]. Some cohort studies have shown that PLWHA on effective ART with viral suppression have COVID‐19 outcomes comparable to HIV‐negative individuals [16, 17].

The type, adherence, and duration of ART regimens may influence the immune response to SARS‐CoV‐2, with integrase inhibitor‐based regimens potentially associated with lower COVID‐19 severity [18]. COVID severity is categorized by World Health Organization as asymptomatic, mild (no pneumonia), moderate (clinical or radiologic pneumonia), severe (respiratory distress or low oxygen saturation), and critical disease requiring intensive support, providing a standardized base for comparing outcomes among PLWHA [16, 19–21].

HIV‐driven immune dysregulation, including alterations in CD4/CD8 counts, B cell function, and cytokine production, can influence susceptibility to SARS‐CoV‐2 infection and disease outcomes [16, 22]. While lower CD4 counts have been linked to higher COVID‐19 severity, some studies suggest that chronic immune activation may paradoxically confer partial protection [23, 24]. Additionally, HIV‐related comorbidities such as hypertension, diabetes, tuberculosis, and cancer further increase COVID‐19 severity, although early detection, effective management, and routine follow‐up may mitigate this risk [19, 20]. Vaccination remains protective, though responses can be weaker at low CD4 counts [25]. Limited Kenyan data suggest fewer diagnoses than expected, implicating ART, comorbidity control, immune dysregulation, vaccination, and genetics [26, 27].

We therefore conducted a cross‐sectional survey to address this gap by evaluating COVID‐19 vaccination status, clinical characteristics, and self‐reported outcomes among people living with HIV (PLWHA) in Kenya. We assessed how ART adherence, duration, and comorbidities relate to perceived risk and vaccine outcomes, treating laboratory biomarkers as exploratory subset observations.

## 2. Methods

### 2.1. Study Design

We conducted an exploratory, cross‐sectional study among PLWHA in Kenya to simultaneously assess clinical characteristics, self‐reported histories, and COVID‐19 outcomes within the study period. While general ART regimen associations were previously noted [28], this paper uniquely evaluates ART adherence and missed doses, vaccination patterns and adverse events, metabolic and comorbidity profiles, and health‐seeking biases.

### 2.2. Study Period, Sites, and Population

Data collection and retrospective record reviews were carried out between 2021 and 2024 across selected Kenyan health facilities located in high HIV‐prevalence regions: Kisumu County Referral Hospital (Kisumu), Rift Valley Provincial General Hospital (Nakuru), and Mariakani and Malindi Sub‐County Hospitals (SCHs, Kilifi). The study population comprised consenting PLWHA aged 18 years who were actively receiving ART.

### 2.3. Sample Size and Sampling

The study targeted an initial sample size of 420 PLWHA, recruited proportionately across participating facilities using systematic sampling. Following data collection, 117 records were excluded due to incomplete questionnaire responses (*n* = 74) or missing clinical history (*n* = 43), resulting in a final analytical sample of 303 participants. Sensitivity checks indicated no systematic demographic differences between included and excluded records.

### 2.4. Data Collection

Quantitative data were obtained through structured questionnaires and abstraction forms capturing demographics, clinical history, ART use, comorbidities, viral load, CD4/CD8 counts, and COVID‐19 vaccination status. Laboratory testing included RT‐PCR using the Thermo Fisher TaqPath™ Kit (LOD ∼10–50 copies/reaction) and Cepheid GeneXpert Xpert® Xpress assay (LOD ∼300–500 copies/mL), and ELISA for SARS‐CoV‐2 antibodies [29]. Infection history was primarily categorized based on self‐reported clinical diagnoses and symptoms, supplemented by retrospective laboratory records (RT‐PCR and ELISA) where available. To minimize recall bias, self‐reported cases were cross‐referenced with health facility registry logs when accessible.

### 2.5. Ethical Considerations

Approval was obtained from the Kenya Medical Research Institute Scientific Ethical Review Unit (Ref: KEMRI/SERU/CVR/XXX/4447) and County Health Departments, while a research permit was issued by the National Council for Science, Technology and Innovations (NACOSTI). Written informed consent was obtained for the prospective follow‐up.

### 2.6. Statistical Analysis

Data were analyzed using IBM SPSS Statistics Version 25 (IBM Corp., 2017). Categorical variables were reported as frequencies and proportions. Continuous variables were summarized as mean ± SD or median (IQR) depending on distribution. Associations between ART, immune markers, vaccination, and COVID‐19 outcomes were assessed using Chi‐square or Fisher’s exact tests. *p* < 0.05 was considered statistically significant. Analyses involving CD4 count and viral load were conducted using available cases, as the mechanism for missing laboratory data was not formally assessed and nonrandom missing data cannot be ruled out.

## 3. Results

### 3.1. Sociodemographic Characteristics

Table 1 presents the sociodemographic characteristics of 303 adults living with HIV attending three high HIV‐prevalence health facilities in Kenya (Kisumu County Referral Hospital, Malindi/Mariakani SCH, and Nakuru County Provincial General Hospital) from 2021 to 2023.

**TABLE 1:** Sociodemographic characteristics (*N* = 303).

Characteristic	Frequency [*n* (%)]
*Health facility*	
Kisumu County Referral Hospital	147 (48.5)
Malindi/Mariakani SCH—Kilifi County	66 (21.8)
Nakuru County Provincial General Hospital	90 (29.7)

*Sex*	
Females	214 (70.6)
Males	89 (29.4)

*Age group in years*	
15–24 (early working age)	24 (7.9)
25–54 (prime working age)	234 (77.2)
55–64 (mature working age)	35 (11.6)
65+ (elderly)	10 (3.3)

*Marital status*	
Single	73 (24.1)
Married (monogamous)	121 (39.9)
Married (polygamous)	21 (6.9)
Separated	28 (9.2)
Divorced	7 (2.3)
Widowed	53 (17.5)

*Education*	
No education	32 (10.6)
Primary school incomplete	51 (16.8)
Primary school complete	74 (24.4)
High school incomplete	43 (14.2)
High school complete	57 (18.8)
Tertiary institution	46 (15.2)

*Occupation in the last 12 months*	
Unemployed/student	61 (20.1)
Retired	4 (1.3)
Farmer	8 (2.6)
Employed	79 (26.1)
Self‐employed	66 (21.8)
Business	85 (28.1)

*Living arrangements*	
Alone	44 (14.5)
Spouse	28 (9.2)
Immediate family	211 (69.6)
Other (aunt, friend, guardian, employer)	6 (2.0)
Not indicated	14 (4.6)

*Note:* Percentages may not sum to 100 due to rounding; “Other” includes aunt, friend, guardian, and employer.

### 3.2. ART

Table 2 presents the ART regimen, duration, changes, adherence, and reasons for missed doses among 303 participants from 2021 to 2023. Most participants were on first‐line regimens (90.8%), predominantly TDF/3TC/DTG or TLD (72.9%). Nearly half had been on ART for more than 10 years. Most participants reported taking ART once daily (94.1%). Among them, 20.1% missed at least one dose, commonly due to forgetfulness, travel, or work‐related reasons.

**TABLE 2:** Antiretroviral therapy (*N* = 303).

Characteristic	Frequency [*n* (%)]
*Regimen*	
First line (AF2E)	48 (15.8)
TDF/3TC/DTG	221 (72.9)
TLD and CPT co‐treatment	3 (1.0)
TDF/3TC/EFV, TLE, or ABC/3TC/DTG	3 (1.0)
Second line [TDF/3TC/ATV/r]	8 (2.6)
TDF/3TC/LPV/r	4 (1.3)
ABC/3TC/ATV/r	3 (1.0)
AZT/3TC/ATV/r	13 (4.3)

*Regimen*	
First line	275 (90.8)
Second line	28 (9.2)

*Duration of ARV use*	
Short term (≤ 3 years)	38 (12.5)
Medium term (> 3–5 years)	29 (9.6)
Long term (> 5–10 years)	99 (32.7)
Long term (> 10 years)	137 (45.2)

*Ever changed ARVs?*	
Yes	253 (83.5)
No	50 (16.5)

*Reasons for changing ARVs (N = 253)*	
Government directive/optimization	185 (73.1)
New drug available/substitution/switch	28 (11.1)
Treatment failure	15 (5.9)
Virological failure	4 (1.6)
Out of stock	7 (2.8)
Others #1	4 (1.6)

*Number of times ARVs have been changed (N = 253)*	
1	216 (85.4)
2	22 (8.7)
3+	15 (5.9)

*How often are ARVs taken?*	
Once	285 (94.1)
Twice	18 (5.9)

*Number of missed ARV doses*	
None	242 (79.9)
1	31 (10.2)
2	16 (5.3)
3+	14 (4.6)

*Reasons for missing daily ARV dose (N = 61)*	
None/no reason given	9 (14.8)
Forgot to take ARVs	15 (24.6)
Traveled and forgot ARVs	10 (16.4)
Busy with work	9 (14.8)
Stigmatization	4 (6.6)
Stress/family stress	3 (4.9)
Domestic issues/violence	3 (4.9)
Sick or unwell	3 (4.9)
Others #2	5 (8.2)

*Note:* Others #1: Toxicity (1 case), Restart after lost to follow‐up (1), Anemia & neuropathy (1), Enlargement of breasts (1). Others #2: Missing appointment, forgetting appointment dates, Ran out of drugs, Pill burden, Time inconvenience (1 each), Percentages may not sum to 100 due to rounding; TLD: Tenofovir disoproxil fumarate, Lamivudine, and Dolutegravir (TLD): first‐line antiretroviral therapy (ART) regimen: *T* = Tenofovir disoproxil fumarate (TDF), *L* = Lamivudine (3TC) and *D* = Dolutegravir (DTG), So, TLD = TDF + 3TC + DTG; CPT: Cotrimoxazole co‐treatment.

### 3.3. Comorbidity and Treatment History

Table 3 presents comorbid conditions, recent flu‐like illnesses, symptoms of chronic lung disease, COVID‐19 history and severity, and key metabolic indicators: body mass index (BMI), waist circumference, and fasting blood glucose, among 303 participants from 2021 to 2023.

**TABLE 3:** Comorbidity, respiratory illnesses, and nutrition status of participants (*N* = 303).

Characteristic	Frequency [*n* (%)]
Comorbidity profile	
Ever suffered from any comorbidity	31 (10.2)
Types of comorbidities (*N* = 31)	
Hypertension	10 (32.3)
Tuberculosis	6 (19.4)
Diabetes	3 (9.7)
Asthma	2 (6.5)
Malaria	2 (6.5)
Cold/flu (previous significant episodes)	3 (9.7)
Others^∗^	7 (22.6)
Recent flu‐like illnesses and COVID‐19 history	
When last had a flu episode (months ago) (*N* = 251)	
< 1 month	83 (33.1)
1–< 3 months	86 (34.3)
3–< 6 months	39 (15.5)
≥ 6 months	43 (17.1)
COVID‐19‐like symptoms (*N* = 244)	
Cough	194 (79.5)
Fever	187 (76.6)
Sore throat	182 (74.6)
Fatigue	178 (73.0)
COVID‐19‐like symptoms	137 (56.1)
How flu was managed (*N* = 244)	
Self‐medication	182 (74.6)
Herbal therapy	11 (4.5)
Hospital admission	4 (1.6)
Other	47 (19.3)
COVID‐19 history	
Ever had COVID‐19 infection	28 (9.2)
How COVID‐19 was determined (*N* = 28)	
Laboratory testing	6 (21.4)
Signs and symptoms	22 (78.6)
COVID‐19 infection severity (*N* = 28)	
Mild	25 (89.3)
Moderate	3 (10.7)
COVID‐19 management (*N* = 28)	
Home‐based care	20 (71.4)
Hospitalized	7 (25.0)
Self‐limiting illness	1 (3.6)
History of chronic lung disease	29 (9.6)
Symptoms among those with chronic lung disease (*N* = 29)	
Difficulty breathing	26 (89.7)
Cough	24 (82.8)
Chest pain	23 (79.3)
Fever	21 (72.4)
Fatigue	1 (3.4)
Nutrition and metabolic indicators	
Ever had obesity (clinical history)	23 (7.6)
Body mass index (BMI) (*N* = 157)	
Underweight (< 18.5 kg/m^2^)	14 (8.9)
Normal (18.5–< 25)	86 (54.8)
Overweight (25–< 30)	32 (20.4)
Obese (≥ 30)	25 (15.9)
Abdominal fat risk (waist circumference) (*N* = 73)	
Low risk	49 (67.1)
Increased risk	6 (8.2)
High risk	18 (24.7)
Fasting blood sugar (*N* = 94)	
< 5.6 mmol/L	88 (93.6)
5.6–6.9 mmol/L	3 (3.2)
≥ 7 mmol/L	3 (3.2)

^∗^Others include arthritis, back pain, Kaposi sarcoma, kidney disease, hypotension, peptic ulcer disease, pneumonia (1 case each); percentages may not sum to 100 due to rounding.

### 3.4. HIV‐Immune Dysregulation, Metabolic Status, and Hospitalization History of Participants

Table 4 presents CD4 T‐cell counts, HIV viral load, BMI, fasting blood sugar levels, hospitalization history, and duration of and reasons for hospitalization among the 303bw participants from 2021 to 2023. CD4 counts were available for only 23 participants, and viral load for 114, limiting statistical power.

**TABLE 4:** HIV‐immune dysregulation, metabolic status, and hospitalization history of participants.

Characteristic	Frequency [*n* (%)]
HIV‐immune markers	
CD4 T‐cell counts (cells/μL) (*N* = 23)	
< 200	3 (13.0)
200–349	6 (26.1)
350–499	6 (26.1)
≥ 500	8 (34.8)
HIV viral load (copies/mL) (*N* = 114)	
< 200	104 (91.2)
200–< 1000	2 (1.8)
≥ 1000	8 (7.0)
Hospitalization history (*n* = 303)	
Ever been hospitalized	89 (29.4)
Never been hospitalized	214 (70.6)
Reason for hospitalization (*N* = 89)	
Childbirth/prolonged labor	16 (18.0)
Malaria	17 (19.1)
Surgery	9 (10.1)
Accident/RTA/fractures	5 (5.6)
Pulmonary TB	9 (10.1)
Pneumonia	7 (7.9)
Diarrhea/stomach upset	4 (4.5)
Typhoid	2 (2.2)
Anemia	2 (2.2)
HIV‐related illness	2 (2.2)
Stroke	2 (2.2)
Chest congestion/pain and cough	2 (2.2)
Others^†^	12 (13.5)
Duration of hospitalization (days) (*N* = 89)	
1–3	36 (40.4)
4–7	36 (40.4)
≥ 8	17 (19.2)

^†^Others include hernia, meningitis, ulcers & tonsils, Kaposi sarcoma, asthmatic problems, prostate cancer, fainting due to hypoglycemia, fever, gastroenteritis, weight loss, missed drug doses, and postoperative hospitalizations; percentages may not sum to 100 due to rounding.

### 3.5. COVID‐19 Infection, Vaccination, Adverse Effects, and Management Practices Among Participants (From 2021 to 2023, *N* = 303)

Table 5 summarizes perceived risk, infection prevalence, disease severity, vaccination status, types and doses of vaccines, and adverse effects. It also contains measures taken to manage COVID‐19 infection. Only 9.2% of participants reported COVID‐19 infection. This may reflect asymptomatic cases, limited testing, or reliance on self‐report.

**TABLE 5:** COVID‐19 infection, vaccination, adverse effects, and management practices among participants (*N* = 303).

Characteristic	Frequency [*n* (%)]
COVID‐19 infection risk and history	
Perceives to be at risk of COVID‐19 infection	170 (56.1)
Not at risk	133 (43.9)
Reason for perceived risk (*N* = 170)	
Death/fear of death	76 (44.7)
Interaction with many people/infected persons	52 (30.6)
Low immunity/HIV+/diabetes	18 (10.6)
Getting infected/infecting others	22 (12.9)
Hospitalization risk	2 (1.2)
Ever had a COVID‐19 infection	28 (9.2)
Management of COVID‐19 infection (*N* = 28)	
Home‐based care	20 (71.4)
Hospitalization	7 (25.0)
Self‐limiting illness	1 (3.6)
Family/close relative tested positive	49 (16.2)
Friend(s) tested positive	100 (33.0)
A family member died from COVID‐19	19 (6.3)
Friend(s) died from COVID‐19	43 (14.2)
COVID‐19 vaccination status	
Ever vaccinated	262 (86.5)
Not vaccinated	41 (13.5)
Vaccine type (*N* = 262)	
AstraZeneca	104 (39.7)
Johnson & Johnson	88 (33.6)
Pfizer	35 (13.4)
Moderna	18 (6.9)
Combination vaccines	15 (5.7)
Number of doses (*N* = 262)	
1 dose	96 (36.6)
2 doses	130 (49.6)
3 doses	36 (13.7)
Believe the vaccine protects from COVID‐19	279 (92.1)
Believe vaccine can worsen health condition	40 (13.2)
Believe COVID‐19 can be acquired via vaccination	30 (9.9)
Knowledge of adverse effects of COVID‐19	
Know the side effects of the vaccine	242 (79.9)
Know some adverse effects	228 (75.2)
Fever/chills	75 (32.9)
Fatigue	87 (38.2)
Headache	91 (39.9)
Dizziness	35 (15.4)
Body ache/pain	50 (21.9)
Numbness/paralysis	29 (12.7)
Swelling/pain at the injection site	38 (16.7)
Malaise/weakness	20 (8.8)
Nausea/vomiting	24 (10.5)
Death	7 (3.1)
Loss of appetite	5 (2.2)
Coughing	4 (1.8)
Others^∗^	13 (5.7)
Practices for managing COVID‐19 infection	
Isolation/quarantine	180 (59.4)
Hospitalization	136 (44.9)
Home‐based care	98 (32.3)
Medication	74 (24.4)
Wearing mask/social distancing/hand hygiene	50 (16.5)
Oxygen therapy	7 (2.3)
Balanced diet/hydration	11 (3.6)
Vaccination	2 (0.7)

^∗^Others reported difficulty breathing, getting very sick, sore throat, rashes, bleeding at the injection site, allergy, lack of sleep, chest tightness, and hallucinations; percentages may not sum to 100 due to rounding.

### 3.6. Associations Between ART, HIV‐Immune Status, COVID‐19 Vaccination, and SARS‐CoV‐2 Infection and Severity From 2021 to 2023

Table 6 shows the association of ART regimen, adherence, duration of use, HIV viral load, CD4 T‐cell counts, and COVID‐19 vaccination with SARS‐CoV‐2 infection and COVID‐19 disease severity among participants.

**TABLE 6:** Associations between ART, HIV‐immune status, COVID‐19 vaccination, and SARS‐CoV‐2 infection and severity.

Characteristic/subgroup	Total (*N* = 303), *n* (%)	SARS‐CoV‐2 infection		*p* value	COVID‐19 disease severity			*p* value
		Yes	No		No infection	Mild	Moderate	
		(*n* = 28)	(*n* = 275)		(*n* = 275)	(*n* = 25)	(*n* = 3)	
ART regimen				0.492				N.V.
First‐line	275 (90.8)	25 (89.3)	250 (90.9)		250 (90.9)	22 (88.0)	3 (100.0)	
Second‐line	28 (9.2)	3 (10.7)	25 (9.1)		25 (9.1)	3 (12.0)	—	
Missed daily ART dose				0.031^∗^				N.V.
Yes	61 (20.1)	10 (35.7)	51 (18.5)		51 (18.5)	8 (32.0)	2 (66.7)	
No	242 (79.9)	18 (64.3)	224 (81.5)		224 (81.5)	17 (68.0)	1 (33.3)	
Duration of ART use				0.384				N.V.
Short‐term (≤ 3 years)	38 (12.5)	4 (14.3)	34 (12.4)		34 (12.4)	4 (16.0)	—	
Medium‐term (> 3–5 years)	29 (9.6)	3 (10.7)	26 (9.5)		26 (9.5)	3 (12.0)	—	
Long‐term (> 5–10 years)	99 (32.7)	5 (17.9)	94 (34.2)		94 (34.2)	4 (16.0)	1 (33.3)	
Long‐term (> 10 years)	137 (45.2)	16 (57.1)	121 (44.0)		121 (44.0)	14 (56.0)	2 (66.7)	
COVID‐19 vaccination				0.014^∗^				N.V.
Vaccinated	262 (86.5)	28 (100.0)	234 (85.1)		234 (85.1)	25 (100.0)	3 (100.0)	
Unvaccinated	41 (13.5)	—	41 (14.9)		41 (14.9)	—	—	
HIV viral load (*n* = 114)		(*n* = 7)	(*n* = 107)	0.484	(*n* = 107)	(*n* = 5)	(*n* = 2)	N.V.
< 200 copies/mL (LDL)^∗^	104 (91.2)	6 (85.7)	98 (91.6)		98 (91.6)	5 (100.0)	1 (50.0)	
≥ 200 copies/mL	10 (8.8)	1 (14.3)	9 (8.4)		9 (8.4)	—	1 (50.0)	
CD4 T‐cell count (*n* = 23)		(*n* = 3)	(*n* = 20)	0.751	(*n* = 20)	(*n* = 3)	(*n* = 0)	N.V.
< 200 cells/μL	3 (13.0)	—	3 (15.0)		3 (15.0)	—	—	
200–500 cells/μL	8 (34.8)	1 (33.3)	7 (35.0)		7 (35.0)	1 (33.3)	—	
> 500 cells/μL	12 (52.2)	2 (66.7)	10 (50.0)		10 (50.0)	2 (66.7)	—	

*Note:* N.V. indicates that chi‐square conditions for valid statistical testing were not met. LDL = Less than detectable limit; N.V. = Chi‐square not valid (conditions for test not met); percentages may not sum to 100 due to rounding. CD4 counts were available for 23 (7.7%) participants and viral load for 114. Due to these extensive missing records and the low absolute number of reported COVID‐19 cases (*N* = 28), cell counts in several strata of the Chi‐square and Fisher’s exact tests were highly limited, precluding a robust multivariable analysis.

### 3.7. COVID‐19 Experiences

Figure 1 shows that less than one‐fifth (16.2%) of family members/close relatives had tested positive for COVID‐19, while 33.0% knew of any nonrelative who had tested positive for COVID‐19. Only 6.3% had a family member who died from COVID‐19 infection, and 14.2% had a friend(s) who died from COVID‐19 infection.

**FIGURE 1:**
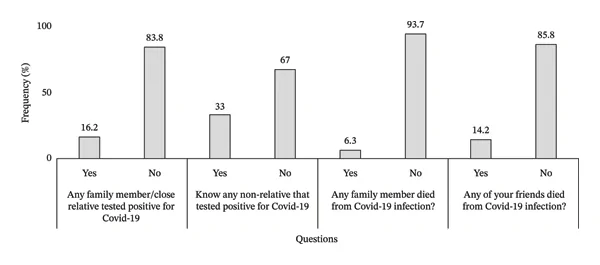
COVID‐19 experiences (*N* = 303).

## 4. Discussion

This study is a comprehensive assessment of sociodemographic characteristics, immune status, comorbidities, metabolic indicators, and COVID‐19 outcome among participants in three low‐income and HIV prevalent regions of Kenya. The association between HIV, ART, comorbidities, and COVID‐19 in a sub‐Saharan African context is therefore detailed. The low reported incidence (9.2%) may reflect limited testing, asymptomatic cases, or reliance on self‐reported infection history, as reported in a study [30].

Most participants were female (70.6%) in their prime working age group (25–54 years), consistent with other studies’ trend in the prevalence of HIV, where women are disproportionately affected due to biological and sociocultural factors [31]. A relatively socioeconomic active cohort which may be associated with a health‐seeking behavior and ART adherence [32] was established among those with a higher level of education (high school or above in 34%) and self‐employment/business activity (49.9%). Household setup included immediate family (69.6%), which can be associated with potential social support structures that can enhance treatment adherence [33].

Majority of the participants were on first‐line ART regimens (90.8%), primarily TDF/3TC/DTG. This is an indication reflecting Kenya’s national guidelines for ART optimization [34]. Long‐term ART use (> 10 years in 45.2%) suggests sustained engagement with HIV care, aligning with studies showing improved viral suppression and immune recovery with prolonged therapy [35]. Similarly to a cohort reported in a sub‐Saharan African study [36], forgetfulness, work, and travel were associated with a missed dose among 20.1% despite high adherence (79.9%). Missed doses were significantly associated with SARS‐CoV‐2 infection (*p* = 0.031), hence the importance of strict adherence to maintain immune protection against opportunistic infections and COVID‐19 as reported elsewhere [35].

Comorbidities were reported by only 10.2%, with hypertension (32.3%) and tuberculosis (19.4%) being the most frequent. The low comorbidity burden contrasts with high‐income settings where morbidity among PLWHA is more prevalent [37]. We established largely normal limits of metabolic indicators, where 62.2% had a normal BMI and 93.6% had normal fasting glucose, although a few participants (12.2%) were obese. There was an increased number of persons with abdominal fat accumulation (24.7%), consistent with the literature linking long‐term ART exposure and weight gain [38, 39]. These metabolic changes may be influenced by long‐term ART exposure, particularly integrase inhibitor‐based regimens, and may also interact with immune status, as higher CD4 counts and viral suppression can modulate metabolic outcomes in PLWHA. Some participants (9.6%) reported chronic lung disease symptoms, highlighting a need for ongoing respiratory health surveillance [40].

Although not all participants had immune marker reports, those available indicated that 34.8% had CD4 counts ≥ 500 cells/μL, and 91.2% of participants with viral load data were suppressed, reflecting effective ART‐mediated viral control. Hospitalization was reported among 29.4% of participants, predominantly for malaria, childbirth, and tuberculosis. This is a reflection of the burden of infectious and reproductive health challenges, especially in malaria‐endemic areas like our study regions. Duration of hospitalization was reported to be typically short (≤ 7 days for 80.8% of cases), suggesting manageable clinical outcomes despite HIV infection [41].

A minimal number of participants (9.1%) reported COVID‐19 infection, with 89.3% experiencing mild disease managed predominantly through home‐based care, aligning with national guidelines [34]. Rather than a true epidemiological drop, this low reported incidence likely reflects testing access limitations, under‐reporting of transient symptoms, high asymptomatic prevalence, and reliance on self‐reported histories over universal laboratory testing. Consequently, formal evaluation favored individuals presenting with moderate to severe symptoms.

Despite low overall COVID‐19 rates, high perceived efficacy (92.1%), and strong adverse‐effect awareness, infection was significantly linked to missing ART doses (*p* = 0.031). While a significant association was observed between vaccination status and reported infection (*p* = 0.014) with cases occurring among vaccinated individuals, this may reflect ascertainment or healthcare‐seeking bias. Vaccinated participants had higher healthcare engagement, increasing their likelihood of undergoing routine testing [42–44]. Due to the cross‐sectional design, small infection count, self‐reported history, unknown exposure timing, and differential testing, vaccine effectiveness could not be reliably estimated, underscoring that high vaccine coverage and ART adherence remain vital for risk mitigation.

ART regimen, duration, and viral load were not significantly associated with COVID‐19 infection or severity, suggesting that well‐controlled HIV may not increase susceptibility when patients adhere to ART and are virally suppressed [45]. Missed ART doses, however, were linked to higher infection rates, highlighting the importance of continuous adherence. These findings align with recent studies from Kenya and sub‐Saharan Africa showing that well‐managed HIV does not worsen COVID‐19 outcomes and have implications for health policy in the region [46].

Immune markers showed no significant association with COVID‐19 outcomes. However, this bivariate analysis was severely underpowered due to extensive missing records (CD4 data missing for 92.4% of the analyzed sample), a major constraint. Hence, a true clinical relationship cannot be definitively evaluated. The high rate of missing laboratory data highlights a critical systemic challenge in retrospective data retrieval and continuous biomarker monitoring in this clinical setting. Consequently, our findings primarily reflect self‐reported clinical histories rather than laboratory‐confirmed immunological status.

This study highlights the need for sustained ART adherence support, ongoing metabolic and respiratory health monitoring, and continued promotion of COVID‐19 vaccination among PLWHA. These findings are relevant for health policy and other sub‐Saharan African countries with a similar HIV burden, emphasizing integrated HIV and pandemic preparedness strategies [45, 47, 48]. These findings also support integrating vaccination campaigns and ART adherence support into routine HIV care in sub‐Saharan Africa.

## 5. Conclusions

In this Kenyan cohort, long‐term first‐line ART use was associated with good viral suppression and low rates of preventable comorbidities and hospitalizations, though limited CD4 data precluded definitive assessment of immune recovery. Reported SARS‐CoV‐2 infections were low and generally mild; however, emerging metabolic risks (overweight, obesity, and altered fasting blood glucose) highlight the critical need to integrate routine metabolic monitoring into standard HIV care. These epidemiological findings must be interpreted cautiously due to significant missing health records, self‐reported symptom data, and inherent recall, testing, and health‐seeking biases. Notably, the occurrence of all reported infections exclusively among vaccinated participants likely reflects healthcare‐seeking and testing bias rather than vaccine failure, as vaccinated individuals had higher healthcare engagement and routine screening. Additionally, conclusions regarding immune markers are severely constrained by low statistical power, as CD4 counts (*n* = 23) and viral load data (*n* = 114) were available for only a small subset of participants.

## 6. Future Research Recommendations

To overcome reliance on retrospective logs and self‐reports, future prospective studies should incorporate universal laboratory confirmation of SARS‐CoV‐2 infection (e.g., real‐time RT‐PCR) and longitudinal CD4/CD8 assessment to rigorously track immune trajectories and asymptomatic infection. Separately, rigorous evaluation of vaccine effectiveness in this population will require specific methodological frameworks, including reliable documentation of vaccination status, dose numbers, and timing, along with appropriate comparison groups and adjustments for key potential confounders.

### 6.1. Public Health and Policy Frameworks

Maintaining optimal clinical outcomes among PLWHA during respiratory pandemics requires national health policies focused on uninterrupted care and prevention. Policy frameworks must guarantee strict, continuous ART adherence to prevent transient immunosuppression while sustaining high primary and booster vaccination coverage across all care networks.

### 6.2. Take‐Home Message

Sustained ART adherence, coupled with COVID‐19 vaccination, proactive metabolic monitoring, and structured adherence support, drives favorable clinical outcomes in PLWHA. HIV care programs must formally integrate routine vaccination campaigns and adherence infrastructure to optimize long‐term health outcomes.

### 6.3. Limitations

Study limitations include extensive missing data in institutional records for baseline immunological biomarkers—specifically CD4 cell counts and viral load data—which constrained statistical power and requires immune dysregulation findings to be interpreted strictly as exploratory. Relying on self‐reported clinical histories, symptoms, and existing records rather than universal laboratory confirmation introduced recall bias along with testing and ascertainment bias, as vaccinated individuals may have had higher healthcare utilization and screening frequency, leading to systematic under‐detection of mild or asymptomatic cases among less healthcare‐engaged or unvaccinated individuals. Additionally, the cross‐sectional design precludes causal mapping between ART regimens, vaccination timelines, and long‐term clinical immunity.

## Author Contributions

James Kinoti Njue, Lihana Wekesa, Alex Maiyo, Zakayo Maingi Mwangi, Dorothy Kagendo, and Raphael Mwamtsi Lwembe designed the study, interpreted the data, and wrote the main manuscript text. All authors reviewed the final manuscript.

## Funding

No funding was received for this manuscript.

## Disclosure

The funding institution had no role whatsoever in designing the study, sample and data analysis, or writing of the manuscript.

## Conflicts of Interest

The authors declare no conflicts of interest.

## Data Availability

The datasets generated during the study are not publicly available due to privacy and ethical restrictions regarding patient clinical records, but are available from the corresponding author upon reasonable request.
